# Evaluation of serum biomarkers for detection of preeclampsia severity in pregnant women

**DOI:** 10.12669/pjms.344.14393

**Published:** 2018

**Authors:** Maryam Kasraeian, Nasrin Asadi, Homeira Vafaei, Tarlan Zamanpour, Hadi Raeisi Shahraki, Khadije Bazrafshan

**Affiliations:** 1 Maryam Kasraeian, M.D. Associate Professor of Prenatalogy. Maternal-Fetal Medicine Research Center, Shiraz University of Medical Sciences, Shiraz, Iran; 2 Nasrin Asadi, M.D. Associate Professor of Prenatalogy. Maternal-Fetal Medicine Research Center, Shiraz University of Medical Sciences, Shiraz, Iran; 3 Homeira Vafaei, M.D. Associate Professor of Prenatalogy. Maternal-Fetal Medicine Research Center, Shiraz University of Medical Sciences, Shiraz, Iran; 4 Tarlan Zamanpour, M.D. Maternal-Fetal Medicine Research Center, Fellowship Perinatology Ward, Shiraz University of Medical Sciences, Shiraz, Iran; 5 Hadi Raeisi Shahraki, PhD. Department of Biostatistics School of Medicine, Shiraz University of Medical Sciences, Shiraz, Iran; 6 Khadije Bazrafshan, MSC. Maternal-Fetal Medicine Research Center, Shiraz University of Medical Sciences, Shiraz, Iran

**Keywords:** Biomarker, Preeclampsia, Pregnancy, Severity

## Abstract

**Objectives:**

To determine serum biomarkers in detection of preeclampsia severity among pregnant women.

**Methods:**

Among 450 pregnant women with various severity of preeclampsia, serum biomarkers ofaspartate aminotransferase (AST), alanine aminotransferase (ALT), lactate dehydrogenase (LDH), hemoglobin (Hb), platelet count (PLT), uric acid, direct bilirubin, total bilirubin, creatinine, and alkaline phosphatase were compared using area under the Receiver operating characteristic (ROC) curve and Area Under the Curve (AUC).

**Results:**

The mean age of women was 30.63±6.43 years and with mean gestational age of 34.69±3.97 weeks. The mean level of LDH, ALT, uric acid, and creatinine were significantly higher in the women with severe type of preeclampsia compared to those with mild type. LDH level had ROC and AUC of more than 0.80, with highest sensitivity, and moderatespecificityin comparison to other markers.

**Conclusion:**

Biomarkers such as ALT, uric acid, and LDH were shown to be prognostic in detection of theseverity of preeclampsia. LDH was demonstrated to significantly be a better prognostic test in detection of preeclampsia severity.

## INTRODUCTION

One of the important conditions in pregnant women is hypertensive disorder with serious maternal and fetal complications.^1^ Among hypertensive disorders, preeclampsia is one of the most important life threatening one for both mothers and neonates worldwide, with 10–15% of the 500,000 maternal deaths each year.^2^,^3^ Routine investigation of women with preeclampsia includes determination of liver function test (LFT) including aspartate aminotransferase (AST), alanine aminotransferase (ALT), lactate dehydrogenase (LDH), bilirubin, and albumin levels. There are controversies for association between these parameters and severity of preeclampsia in literature.^4^

Mean arterial blood pressure, uterine vessels ultrasound,^5^ serum calcium level^6^, serum uric acid level^4^,^6^, LDH^7^, angiogenesis factors such as placental growth factor (PIGF) and vascular endothelial growth factor (VEGF),^2^,^8^ lipid profile,^8^ and glucose tolerance test^9^ were previously studied to evaluate the association between these parameters and severity of preeclampsia. In UK, several guidelines were presented for early diagnosis based on assessment of maternal risk factors; including a screening strategy based on maternal history and characteristics by the National Institute of Clinical Excellence (NICE) predicting less than 30% of those developing preeclampsia.^10^ So there is serious need to assess this association. The present study was undertaken to compare serum biomarkers in pregnant women with different severities of preeclampsiaas prognostic ones in maternal and fetal outcomes.

## METHODS

This retrospective study included 450 women with preeclampsia who were referred to Hafez Hospital affiliated to Shiraz University of Medical Sciences from 2005 to 2014. The study was approved by the local Institutional Ethics Review Board (EC-P-9382-8682 dated Feb. 13, 2014). The inclusion criteria for the women with mild preeclampsia were (i) Systolic blood pressure ≥140 mmHg or diastolic blood pressure ≥90 mmHg and (ii) Proteinuria ≥0.3 grams in 24-hour urine specimen.

The inclusion criteria for the women with severe preeclampsia^11^ were:

1. Signs of liver problems (such as vomiting with abdominal pain)
2. Signs of central nervous system problems (such as blurry vision and severe headache)
3. Very high blood pressure (greater than 160 systolic or 110 diastolic)
4. At least twice the normal measurements of certain liver enzymes on blood test
5. Thrombocytopenia
6. Greater than 5g of protein in a 24-hour sample
7. Very low urine output (less than 500mL in 24 hours)
8. Signs of respiratory problems (such as pulmonary edema)
9. Severe fetal growth restriction
10. Stroke

The women with unstable conditions were excluded from the study.Information such as age, gestational age (GA), and serum levels of LDH, hemoglobin (Hb), platelet count (PLT), uric acid, direct bilirubin, total bilirubin, AST, ALT, serum creatinine (SCr), and alkaline phosphatase, were compared between the women with mild and severe preeclampsia.The obtained data were reported as mean±SD. SPSS software (Version 20, Chicago, IL, USA) was used for statistical analysis. Independent T-test,Pearson’s chi-square and Fisher’s exact tests were used to compare the groups. Receiver Operating Characteristic (ROC) curve was drawn to specify the optimal cut-off points of the variables for prediction of preeclampsia. Sensitivity, specificity, and the Areas Under the Curves (AUC) were also calculatedwith 95% confidence intervals. A P-value<0.05 was considered as statistically significant.

## RESULTS

Out of the 450 women, 180 and 270 ones wereassignedto the mild and severe preeclampsia groups, respectively. Among the 450 births, 221 neonates were male, 217 were female, and 12 were twin male-female neonates. The mean age of pregnant women was 30.63±6.43 years with the mean GA of 34.69±3.97 weeks. Out of the 12 women with twin pregnancies, 11 (91.67%) showed severe preeclampsia (P=0.03). No significant association was noted between the neonates’ sex and severity of preeclampsia (P=0.49).The comparison of serum biomarkers in pregnant women with mild and severe preeclampsia was presented in Table-I.

**Table-I:** Paraclinical parameters of the pregnant women with severe and mild preeclampsia.

Parameter	Severity of preeclampsia	Mean± SE	P-value†
Age (year)	Mild	30.16±5.96	0.24
	Severe	30.89±6.76	
GA (month)	Mild	37.73±2.27	<0.001
	Severe	32.67±3.52	
LDH (IU/L)	Mild	337.89±173.15	<0.001
	Severe	556.41±193.02	
Hb (g/dL)	Mild	11.8±1.62	0.89
	Severe	11.78±1.8	
PLT (×10^9^.L^-1^)	Mild	191.18±49.06	0.019
	Severe	179.08±56.35	
Uric acid (mg/dL)	Mild	5.43±1.2	<0.001
	Severe	6.2±1.4	
Direct bilirubin (mg/dL)	Mild	0.23±0.08	0.415
	Severe	0.24±0.09	
Total bilirubin (mg/dL)	Mild	0.6±0.22	0.86
	Severe	0.6±0.18	
ALT (IU/L)	Mild	18.78±30.38	0.001
	Severe	32.99±54.07	
AST (IU/L)	Mild	32.45±50.8	0.07
	Severe	43.54±73.63	
SCr (mg/dL)	Mild	0.84±0.27	0.017
	Severe	0.9±0.26	
Alkaline phosphatase (IU/L)	Mild	333.94±120.61	0.35
	Severe	321.42±147.74	

† Independent T-test, ALT: alanine transaminase, AST:aspartate transaminase, SCr: serum creatinine, GA:gestational age, Hb: hemoglobin, LDH: lactate dehydrogenase, PLT: platelet count, SE: standard error

The two groups were significantly different for GA, LDH, PLT, uric acid, ALT, and SCr.The mean GA of women with mild preeclampsia was higher compared to those with severe preeclampsia (P<0.001). The mean level of PLT was also higher among the mild cases of preeclampsia in comparison to those with severe preeclampsia (*P*=0.019). The mean level of LDH, ALT, uric acid, and SCr were significantly higher in the women with severe type preeclampsia compared to those with mild type.

**Fig.1:**
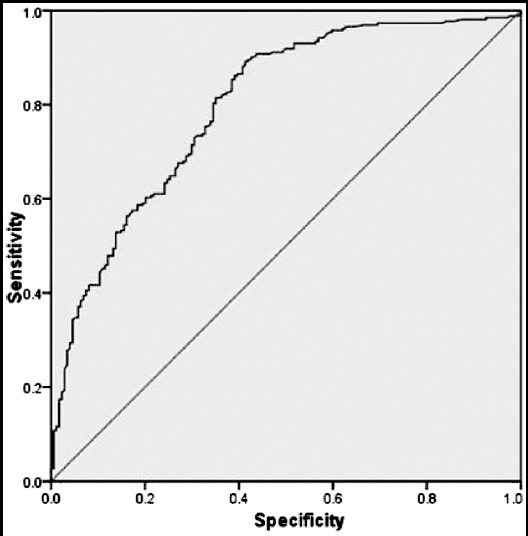
Receiver operating characteristic (ROC) curve for lactate dehydrogenase (LDH).

The ROC curve for LDH is shown in Fig.1.Findings for variables with higher AUC are presented in Table-II, but for variables with AUC<0.6 were notshown. The LDH level had ROC and AUC >0.80, withthe highest sensitivity, and moderatespecificityin comparison to other tests. ALT,AST, and uric acid denoted to a moderatesensitivity and specificity, while SCr had the highest specificity and the lowest sensitivity among the variables (Table-II).

**Table-II:** The variables with high area under curve (AUC). The data are presented as point estimation with 95% confidence interval.

Paraclinical parameters	AUC	Cut-off point	Sensitivity	Specificity
LDH	0.805 (0.765-0.842)	336	89.62 (86.22-93.02)	59.3 (51.6-66.7)
Uric acid	0.666 (0.618-0.712)	5.53	68.67 (62.5-74.4)	58.54 (50.6-66.2)
ALT	0.657 (0.609-0.702)	14.33	62.16 (56-68.1)	62.73 (54.8-70.2)
AST	0.604 (0.556-0.651)	0.25	57.92 (51.6-64)	60.49 (52.5-68.1)
SCr	0.604 (0.555-0.652)	0.96	29.64 (24.1-35.7)	89.1 (83.1-93.5)

ALT: alanine transaminase,AST:aspartate transaminase, SCr: serum creatinine,LDH: lactate dehydrogenase

## DISCUSSION

Maternal complications, such as proteinuria, eclampsia, severe hypertension, hemolysis, abnormal LFTs, and low platelet count can result from preeclampsia.^12^,^13^ Some biomarkers have also been reported to be significantly associated with preeclampsia during pregnancy.^14^ Martin et al.^15^ proposed a protocol for quick hazard assessment of severe preeclampsiashortlyafter meeting study admission criteria evaluating symptoms and levels of AST, ALT, LDH, uricacid, proteinuria, and creatinine.The differences in SCr level between the normal pregnant women and those suffering from preeclampsia were shown not to be significant.^16^

Our results were in line with the study presented an increased level of SCr in preeclampsia women compared to those with normal pregnancy.^17^ Our findings revealed that SCr test was poorly sensitive and had the lowestpredictive value to differentiate the severity of preeclampsia. Uric acid level, as a marker of oxidative stress, tissue injury, and renal dysfunction, is increased in hypoxia and ischemia of the placenta.^11^ Thus, measuring serum uric acid may be used to predict preeclampsia and the mean level of uric acid can be associated with the severity of preeclampsia.^18^

Alavi et al.^6^ found a significant difference for serum calcium and uric acid between the normal pregnant women and those with signs of preeclampsia. In 2009, serum uric acid was found to predict maternal complications in management of preeclampsia under realistic assumptions. It was demonstrated that preeclamptic patients with increased serum uric acid values had to undergo induced labor due to their increased risk of complications.^13^ Similarly, our results showed a higher mean level of maternal serum uric acid in women with severe preeclampsia in comparison to those with mild type (P<0.001).

Although the mean level of uric acidwas higher in preeclampsia pregnant in comparison to women with normal pregnancy, uric acid level cannot be used as a suitable index for management of preeclampsia.^19^-^21^ The mean increase in uric acid levelwas variable in preeclampsia patients in different areas that can explain the difference. The diversity in areas, diets, nutrition, and breeds of preeclampsia subjects can be other contributing factors.^22^,^23^Burwick and Feinberg (2013)^24^ found a considerable increase in liver enzymes in patients with preeclampsia and showed that high levels of AST and ALT can be considered to categorize the severity of preeclampsia.^11^

Kozic JR et al.^4^ in 2008 women with preeclampsia investigated LFTresults to predict adverse maternal outcomes and demonstrated that 53% of patients had at least one abnormality in LFT levels denotingto an increased risk of adverse maternal outcomes.In a systematic review article, Thangaratinam S et al.^25^ investigated the accuracy of LFT for predicting adverse maternal and fetal outcomes in women with preeclampsia as the best moderate predictors of maternal and fetal complications in women with preeclampsia. ALT level was significantly higher in the severe cases of preeclampsia compared to the mild ones. Similar to the study by Thangaratinam et al.^25^, our results revealed a moderate sensitivity and specificityfor LFTin differentiation of preeclampsia severity.

Demir SC et al.^26^ indicated a statistically significant relationship between maternal complications and high LDH levels. Moreover, Odendaal HJ et al.^27^ presented that LDH level before delivery was significantly higher in early onset of severe preeclampsia. Jaiswar et al.^7^showed that LDH level significantly elevated in women with preeclampsia and eclampsia. They reportedthat high serum LDH level had a correlation for severity of the disease and poor outcomes in patients with preeclampsia and eclampsia. In the same line, Qublan et al.^28^ revealed that the mean LDH level was 348±76 IU/l in patients with mild preeclampsia and 774±69.61 IU/l in those with severe preeclampsia. They also found a significant association between serum LDH level and severity of preeclampsia. In the present study, the mean LDH level was 337.89±173.15IU/l and 556.41±193.02IU/l in patients with mild and severe preeclampsia, respectively.

Kozic et al.^6^ displayed that LDH level had ROC and AUC > 0.70 and were modestly predictive tests to show adverse maternal outcomes in women with preeclampsia. In agreement with Kozic et al.^4^, our finding demonstrated that LDH level had ROC and AUC > 0.80, with the highest sensitivity, and moderatespecificityin comparison to other tests. The study by Peralta et al.^29^ showed a significant difference between the mild and severe preeclampsia patients and healthy controls regarding LDH level too. In the present study, LDH levelsignificantly raised based on the severity of the disease (*P*<0.001).

### Limitations of the study

It included lack of a normal and healthy control group and lack of attention to the patients’ trimester of pregnancy as there are different strategies in prediction of preeclampsia in each trimester.

## CONCLUSION

We canconclude that ALT, uric acid, and LDH levelscan be predictivefactors in identification and categorization of the severity of preeclampsiain pregnant women, even LDH level had the highest sensitivity and moderate specificity in comparison to other parameters.
